# Radiation-related Adverse Effects of CT-guided Implantation of ^125^I Seeds for Thoracic Recurrent and/or Metastatic Malignancy

**DOI:** 10.1038/s41598-019-51458-5

**Published:** 2019-10-15

**Authors:** Zhe Ji, Yuliang Jiang, Fuxin Guo, Ran Peng, Haitao Sun, Panfeng Wang, Jinghong Fan, Junjie Wang

**Affiliations:** Department of Radiation Oncology, Peking University Third Hospital, Beijing, China

**Keywords:** Radiotherapy, Lung cancer

## Abstract

During radioactive Iodine-125 seed implantation (RISI), Iodine-125 radionuclide is implanted directly into a lesion and kills tumor cells by steadily emitting radiation. In our study, we analyzed the adverse effects of RISI for thoracic malignancy, and investigated the safety, dosage, and adverse effects of RISI for these cases. Between June 2007 and January 2018, 77 patients with thoracic recurrent and/or metastatic tumors who underwent CT-guided RISI were enrolled. Radiation-related adverse effects were analyzed, including pneumonia, esophagitis, hemorrhage, fistula, skin injury, heart injury, and spinal cord injury. We used the Common Terminology Criteria for Adverse Events (CTCAE) v4.03 to evaluate adverse effects and analyzed the relationship between adverse effects and dosimetric parameters of organs at risk (OAR), including D0.1cc, D2cc, Dmean, and V20. The results of the study were as follows: The median follow-up period was 11 months. The median postoperative dose (D90) was 122 Gy (45.7–241.8 Gy). Three patients (3.9%) showed radiation pneumonitis of grade ≥2. Two patients (2.6%) showed radiation-induced esophagitis of grade ≥2. One patient (1.3%) showed an esophageal fistula. Two patients (2.6%) had a tracheal fistula. Five patients (6.5%) had radiation-related skin reactions. One patient (1.3%) reported chest wall pain, while three (3.9%) showed hemoptysis. No patients showed radiation myelitis or cardiotoxicity. The mean D2cc of organs at risk were 165.7 Gy (lung), 10.61 Gy (esophagus), 10.25 Gy (trachea), 18.07 Gy (blood vessel), 12.64 Gy (heart), 14.77 Gy (spinal cord), 17.47 Gy (skin). Dosimetric parameters, such as D0.1cc, D2cc and Dmean, were higher in patients with toxic reactions (above the upper limit of 95% confidence interval among the overall data). Chi-square test showed that skin D0.1cc > 600 Gy, D2cc > 500 Gy, and Dmean >90 Gy were associated with grade ≥2 radiation dermatitis (p < 0.05), but no clear dose-toxicity correlation was found in other OARs. So, we concluded that the overall incidence of toxicity and adverse effects from RISI for the treatment of thoracic tumors is low. The dose-toxicity characteristics have not been fully defined. Doses within the upper limit of the 95% confidence interval may be considered safe. This was a retrospective analysis, and follow-up period was minimal, indicating possible limitations of this study.

## Introduction

Radioactive seed implantation is a type of brachytherapy characterized by the direct implantation of a radionuclide into a lesion to kill tumor cells by steadily emitting radiation. Iodine-125 seed is the most common implanted seed type. The γ-rays of <0.0355 MeV of Iodine-125 seed can inhibit cell proliferation and angiogenesis, induce apoptosis, and kill tumor cells^1,2^. At present, radioactive Iodine-125 seed implantation (RISI) is one of the radical treatments for early-stage prostate cancer^3^.

In addition to its applications in prostate cancer treatment, RISI also plays an important role in the treatment of head and neck, lung, pancreas, rectum, and other cancers^4–7^. RISI is also known as low dose-rate brachytherapy. Like other radiation therapies, the emitted radiation not only kills tumors, but also causes damage to surrounding tissues. However, although the toxicity and adverse effects of RISI, and the dose limits for organs at risk (OAR), in prostate cancer have been widely investigated^3^, studies on thoracic tumors have focused on the clinical efficacy of RISI, regardless of its side effects^5,8–10^ (in these studies, the local control rate was approximately 60–80%, but the toxicity was seldom mentioned). In this study, we retrospectively analyzed the adverse effects of RISI in patients with thoracic recurrent and/or metastatic tumors in order to further clarify the safety of RISI in thoracic tumors.

## Methods

### Clinical information

A total of 77 patients with thoracic recurrent and/or metastatic tumors who received RISI at our center between June 2007 and January 2018 were enrolled in this study. Indications for RISI were as follows: (1) failure to carry out surgery or external radiotherapy; (2) solitary tumor and tumor size ≤6 cm; (3) definitive pathological diagnosis; (4) suitable puncture access: avoiding bones, large blood vessels, and other organs; (5) no bleeding tendency; and (6) good general condition (Karnofsky performance status >70) with expected survival >3 months. The patients’ median age was 61 years (17–88). All patients agreed to participate in this study by providing written informed consent, and the study was approved by the ethics committee of our hospital. This is a retrospective study, all patients received conventional treatment, and all methods were performed in accordance with the relevant guidelines and regulations. The general information for all patients is shown in Table 1. As recommended in our previously published data, the prescribed dose was 110–160 Gy.

**Table 1:** General patient information.

Characteristics	Cases	Percentage
**Sex**		
Male	48	62.3%
Female	29	37.7%
**Type**		
Primary	33	42.9%
Metastatic	44	57.1%
**Location**		
Lung	55	71.4%
Mediastinum	6	7.8%
Chest wall	16	20.8%
**Previous EBRT**		
Yes	49	63.6%
No	28	36.4%

### System planning and seeds information

A brachytherapy treatment planning system (BTPS) (KLSIRPS-3D) was provided by the Beijing University of Aeronautics and Astronautics and Beijing Astro Technology Co., Ltd. Planning system source data originated from the latest official manuscripts of the American Association of Physicists in Medicine (AAPM)^11,12^. The seed model was 6711_1985 (Shanghai GMS Pharmaceutical Co., Ltd.).

### Preoperative planning design

All patients underwent spiral computer tomography (CT) 2 days before surgery. Patients were positioned according to the tumor site and then fixed with vacuum pads and marked with a positioning line on the body surface. CT data were transmitted to a BTPS for preoperative planning design: (a) delineation of the gross tumor volume (GTV) and adjacent OARs; (b) setting the prescribed dose and seed radioactivity; (c) determination of the needle tract for the implanted seed (insertion direction, distribution, and depth); (d) calculation of the seed number and simulation of the spatial distribution of seeds; and (e) calculation of the dose distribution of the target volume and OARs (spinal cord, blood vessels, trachea, and hollow viscera). We optimized the plan to ensure that doses of 90% GTV (D90 of GTV) matched the prescribed doses (110–160 Gy) as closely as possible and the doses of OAR were as low as possible.

### Puncture and implantation of Iodine-125 seeds

**Figure 1:**
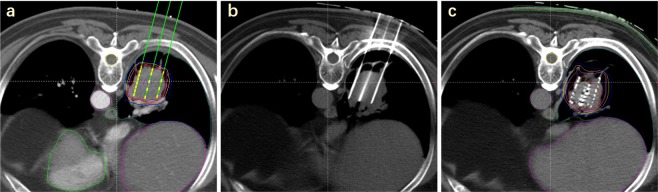
Operation process: (**a**) preoperative planning; (**b**) needle insertion; (**c**) seed implantation.

All patients underwent local infiltration anesthesia and intercostal nerve block. Then, depending on preoperative and intraoperative planning, RISI (with or without the assistance of a three-dimensional printed template) was performed under CT. The seeds spacing referenced the preoperative planning design to within 0.5–1.0 cm. Finally, we observed the actual distribution of seeds and, if necessary, supplemented seeds in real time. Operation processes are shown in Fig. 1. The technical insertion of radiation seed implants followed the expert consensus about radioactive seeds permanent interstitial brachytherapy^1^.

### Postoperative verification of dosimetry

**Figure 2:**
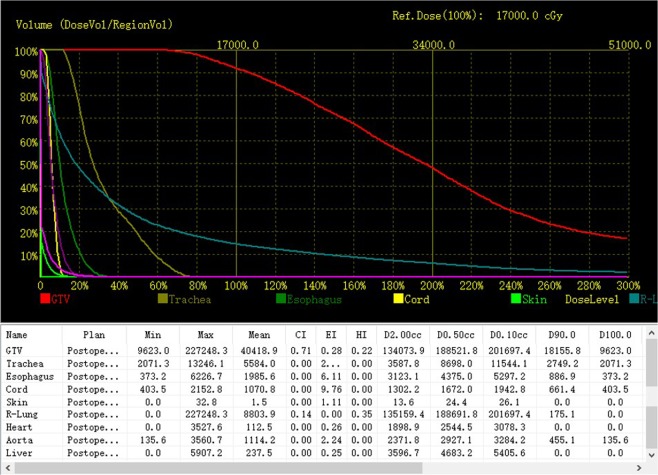
Dosimetric evaluation of GTV and OARs by dose-volume histogram.

On the basis of postoperative CT images, we performed dosimetric evaluations of GTV and adjacent OARs by DVH (dose-volume histogram) (Fig. 2). We used D90 as the evaluation index for GTV. The OARs were evaluated with the parameters used in prostate brachytherapy, including D0.1cc, D2cc, and Dmean (dose to the most exposed 0.1 cc and 2 cc volumes of OARs, and mean dose to OARs)^13,14^. The OARs included the lung, blood vessels, spinal cord, esophagus, trachea, heart, and skin.

### Follow-up examinations

Follow-up assessments were performed at 3, 6, 9, and 12 months postoperatively and every 6 months thereafter. These assessments involved regular outpatient visits and telephone conversations. CT scan was needed to determine any change in tumor status at each of the schedule post-operative visits.

### Parameters under observation

We focused on radiation-related adverse reactions, including pneumonia, esophagitis, hemorrhage, fistula, skin injuries, and spinal cord injuries. The severity of each complication was assessed by physicians according to common terminology criteria for adverse events version 4.03 (CTCAE v4.03) criteria^15^. We also analyzed the relationship between adverse reactions and dosimetric parameters of OARs.

### Statistical analysis

Descriptive statistics were mainly used for statistics of quantity and proportion. Each group of data was represented by median, mean and 95% confidence interval (CI). Chi-square test was used to compare the differences between groups. P < 0.05 was considered statistically significant. The statistical analyses were performed using SPSS 20.0 software (IBM).

### Ethical approval and consent to participate

The study protocol was approved by the Ethics Committee of Peking University Third Hospital. All patients provided written informed consent to participate in this study.

## Results

The median follow-up period was 11 months (1–129.6 months), 49 cases died and 28 cases survived. The median survival time of 49 dead patients was 9.7 months (1–85.2 months). The response rate (complete response + partial response + stable disease described in Response Evaluation Criteria in Solid Tumors v1.1^16^) at 3 months after RISI was 83% (64/77), 7 cases achieved complete response. The median lesion volume was 34.2 cc (1.5–337 cc), the mean number of I-125 seeds was 53 (7–209), the mean number of puncturing needles was 12 (5–26), and the median radioactivity of RISI was 0.63 mCi/23.31 MBq (0.39–0.9 mCi/14.43–33.3 MBq). The median dose (D90) was 122 Gy (45.7–241.8 Gy). All parameters for OARs are shown in Table 2. We also evaluated the affected lung with V20 (0%-64.1%). The median and mean V20 values were 7.26% and 12.55%, respectively.

**Table 2:** Dosimetric parameters of OARs.

	D0.1cc (Gy)				D2cc (Gy)				Dmean (Gy)			
	Median	Mean	95% CI	Range	Median	Mean	95% CI	Range	Median	Mean	95% CI	Range
Lung	579.9	780.9	623.32–938.53	895–1910.09	96.88	165.7	131.70–199.68	3.01–524.23	11.95	22.3	17.31–28.66	0.24–93.14
Esophageal	1.99	13.41	7.81–19.01	0–105.69	1.74	10.61	6.27–14.95	0–85.56	0.29	3.92	2.31–5.52	0–35.57
Trachea	1.88	12.55	6.73–18.36	0–117.22	1.53	10.25	5.77–14.72	0–93.56	0.47	3.5	1.93–5.06	0–35.40
Blood vessels	2.89	26.32	14.07–38.58	0–292.89	2.01	18.07	9.76–26.37	0–193.46	0.34	5.04	2.74–7.35	0–56.70
Heart	5.82	29.77	19.38–40.16	0–188.81	2.77	12.64	8.26–17.01	0–83.81	0.21	1.76	0.98–2.53	0–20.96
Spinal cord	0	23.98	6.44–41.52	0–606.80	0	14.77	6.97–22.57	0–172.22	0	7.87	3.02–12.71	0–114.77
Skin	5.89	30.45	16.08–44.80	0–384.75	4.22	17.47	10.81–24.11	0–148.64	0.9	4.44	2.84–6.04	0–26.90

The incidence of adverse reactions was low: 3 patients (3.9%) had radiation pneumonitis of grade ≥2, including two (2.6%) grade 2 cases and one (1.3%) grade 3 case, all of which occurred within 3 months of RISI. Two patients (2.6%) had radiation esophagitis of grade ≥2, including one (1.3%) grade 2 case and one (1.3%) grade 3 case, which occurred 4 months and 5 months after RISI, respectively. One (1.3%) patient developed an esophageal fistula 5 months after implantation. Two (2.6%) patients had a tracheal fistula, which developed 5 and 9 months after the operation. Five (6.5%) patients had grade 2 radiation-induced skin reactions, all of which occurred within 6 months of implantation. One (1.3%) patient developed chest wall pain within two months. Three (3.9%) patients had hemoptysis, which occurred 1, 6, and 10 months after implantation. None of the patients showed radiation myelitis and cardiotoxicity (Table 3). Chi-square test showed that high skin dose (D0.1cc > 600 Gy, D2cc > 500 Gy, and Dmean >90 Gy) was associated with grade ≥2 radiation dermatitis (p < 0.05), but no dose-toxicity correlation was found in other OARs (Table 4).

**Table 3:** Incidences of side effects.

Side effects	Cases	Percentage
Radiation pneumonitis (grade ≥2)	3	3.9%
Radiation-induced esophagitis (grade ≥2)	2	2.6%
Esophageal fistula	1	1.3%
Tracheal fistula	2	2.6%
Radiation myelitis	0	0.0%
Radiation-induced skin reaction (grade ≥2)	5	6.5%
Chest wall pain	1	1.3%
Hemoptysis	3	3.9%

**Table 4:** Chi-square tests of side effects of organs at risk.

			Radiation Pneumonitis		χ^2^	p
			Grade 0–1	Grade ≥ 2		
Affected lung D0.1cc (Gy)	<60000	n = 39	39 (100%)	0 (0%)	3.204	0.073
	>60000	n = 38	35 (92.1%)	3 (7.9%)		
Affected lung D2cc (Gy)	<10000	n = 40	40 (100%)	0 (0%)	3.375	0.066
	>10000	n = 37	34 (91.9%)	3 (8.1%)		
Affected lung Dmean (Gy)	<1200	n = 40	40 (100%)	0 (0%)	3.375	0.066
	>1200	n = 37	34 (91.9%)	3 (8.1%)		
Affected lung V20 (%)	<7	n = 38	37 (97.4%)	1 (2.6%)	0.32	0.571
	>7	n = 39	37 (94.9%)	2 (5.1%)		
			**Radiation-Induced Esophagitis**		**χ2**	**p**
			**Grade 0–1**	**Grade** **≥** **2**		
Esophagus D0.1cc (Gy)	<200	n = 39	39 (100%)	0 (0%)	2.107	0.147
	>200	n = 38	36 (94.7%)	2 (5.3%)		
Esophagus D2cc (Gy)	<200	n = 39	39 (100%)	0 (0%)	2.107	0.147
	>200	n = 38	36 (94.7%)	2 (5.3%)		
Esophagus Dmean (Gy)	<30	n = 40	40 (100%)	0 (0%)	2.22	0.136
	>30	n = 37	35 (94.6%)	2 (5.4%)		
			**Radiation-Induced Skin Reaction**		**χ2**	**p**
			**Grade 0–1**	**Grade** **≥** **2**		
Skin D0.1cc (Gy)	<600	n = 39	39 (100%)	0 (0%)	5.488	0.019
	>600	n = 38	33 (86.8%)	5 (13.2%)		
Skin D2cc (Gy)	<500	n = 41	41 (100%)	0 (0%)	6.09	0.014
	>500	n = 36	31 (86.1%)	5 (13.9%)		
Skin Dmean (Gy)	<90	n = 39	39 (100%)	0 (0%)	5.488	0.019
	>90	n = 38	33 (86.8%)	5 (13.2%)		
			**Tracheal Fistula**		**χ2**	**p**
			**Without**	**With**		
Trachea D0.1cc (Gy)	<200	n = 39	39 (100%)	0 (0%)	2.107	0.147
	>200	n = 38	36 (94.7%)	2 (5.3%)		
Trachea D2cc (Gy)	<150	n = 38	38 (100%)	0 (0%)	2.001	0.157
	>150	n = 39	37 (94.9%)	2 (5.1%)		
Trachea Dmean (Gy)	<50	n = 39	39 (100%)	0 (0%)	2.107	0.147
	>50	n = 38	36 (94.7%)	2 (5.3%)		
			**Hemoptysis**		**χ2**	**p**
			**Without**	**With**		
Blood vessel D0.1cc (Gy)	<300	n = 39	39 (100%)	0 (0%)	3.204	0.073
	>300	n = 38	35 (92.1%)	3 (7.9%)		
Blood vessel D2cc (Gy)	<200	n = 38	38 (100%)	0 (0%)	3.042	0.081
	>200	n = 39	36 (92.3%)	3 (7.7%)		
Blood vessel Dmean (Gy)	<40	n = 39	39 (100%)	0 (0%)	3.204	0.073
	>40	n = 38	35 (92.1%)	3 (7.9%)		

The patient with grade 3 radiation pneumonitis had no previous external beam radiotherapy (EBRT). The D0.1cc, D2cc, Dmean, and V20 of the affected lung of this patient were 1763.47 Gy, 327.33 Gy, 54.39 Gy, and 41.6%, respectively. One of the two patients with grade 2 radiation pneumonitis had received previous EBRT (50 Gy, 1 year before implantation), and the values of the aforementioned parameters for this patient were 1881.19 Gy, 272.38 Gy, 30.92 Gy, and 5.9%, respectively. The other patient with grade 2 radiation pneumonitis had no previous EBRT, and the corresponding values for the four parameters were 1910.09 Gy, 476.57 Gy, 73.30 Gy, and 23.4%, respectively. One patient with grade 3 radiation esophagitis had a tracheoesophageal fistula at the same time, and had a history of EBRT (56 Gy, 10 months before RISI). The D0.1cc, D2cc, and Dmean of the esophagus were 38.92 Gy, 31.22 Gy, and 11.39 Gy, respectively, in this patient. One patient with grade 2 radiation esophagitis, the above indicators were 105.69 Gy, 85.56 Gy, and 35.57 Gy, respectively. Among the patients with tracheal fistulas, one patient also had esophagitis of grade 3, and the D0.1cc, D2cc, and Dmean of the trachea were 105.32 Gy, 68.88 Gy, and 21.83 Gy, respectively. Another patient had a history of EBRT (70 Gy, 6 years before implantation), and the above indicators were 23.56 Gy, 16.02 Gy and 3.89 Gy, respectively. Five patients with radiation-induced skin reactions of grade 2 also had chest wall lesions. Two of these patients had a history of EBRT (66 Gy, the interval between EBRT and implantation was 9 months and 4 years, respectively). For the skin, the D0.1cc was 83.93–384.75 Gy (median, 125.54 Gy; mean, 190.12 Gy), D2cc was 54.62–151.14 Gy (median, 65.42 Gy; mean, 82.11 Gy), and Dmean was 14.20–26.90 Gy (median, 19.54 Gy; mean, 20.32 Gy). One patient with chest wall pain had a history of EBRT (70 Gy, the interval was 8 months) and the D0.1cc, D2cc, and Dmean of the skin were 251.15 Gy, 148.64 Gy, and 21.51 Gy, respectively. Three patients had low-grade hemoptysis. The first of these patients had received EBRT (50 Gy) 10 years before implantation. The second received EBRT (66 Gy) 1 year before implantation, and the third received EBRT (60 Gy) 4 years before implantation. For the first patient, the D0.1cc, D2cc, and Dmean for blood vessels were 137.45 Gy, 82.96 Gy, and 19.79 Gy. The corresponding values for the second and third patients were 105.22 Gy, 59.60 Gy, and 12.24 Gy and 121.98 Gy, 86.85 Gy, and 36.31 Gy, respectively.

## Discussion

Internationally, RISI is typically used for the treatment of prostate cancer, and studies on the toxicity response of OARs have mainly focused on the bladder, rectum, and urethra^3^. For thoracic tumors, RISI can be used in cases with a positive postoperative incisal margin, cases requiring palliative treatment for advanced lesions, and cases of recurrent lesions after surgery/EBRT and chemotherapy^17^. However, due to the limited application of this technology, few studies have reported the optimal control dose for GTV and the tolerance dose for OARs^17^. The present study provides detailed data related to the toxicity and dose-related parameters of patients treated with ^125^I seed implantation in our department. The findings are of great significance to further understand the safety of this treatment and to guide future studies.

In this study, although most patients had previously undergone chest external beam radiotherapy (EBRT), the incidence of adverse reactions after RISI was still low. Apart from skin reactions (6.5%), the incidence of all other adverse reactions was less than 5%. Except for one case of grade 3 radiation pneumonitis, and three cases of esophageal/tracheal fistula (3.9%), there were no other adverse reactions of grade ≥3. Recent studies have analyzed the toxicity and side effects of RISI in head and neck tumors. Although those studies assessed different OARs, they showed no obvious toxicity or side effects of grade ≥3^18^, demonstrating the safety of RISI.

Radiation pneumonitis is a specific, toxic, adverse effect of thoracic EBRT. Previous studies have reported that the incidence of grade 3 radiation pneumonitis is about 20% for re-irradiation, even with stereotactic radiotherapy^19,20^. In our study, only one of the three patients with radiation pneumonitis of grade ≥2 had previously undergone EBRT. For patients with grade 3 pneumonitis, only the V20 was higher than in other patients (41.6%), while the D0.1cc, D2cc, and Dmean showed no significant differences. In our study, V20 was assessed for the affected lung. However, according to the current standard of external beam radiotherapy, V20 should be evaluated for both lungs. The reasons we did not evaluate both lungs are that the dose of radioactive seeds decayed rapidly, the opposite lung had no substantial dose, and the V20 of both lungs was very low. Therefore, we consider radiation pneumonitis in RISI to be not only dose-dependent, but potentially associated with autoimmune function or infectious pneumonia^21^.

Since the esophagus and trachea are serial organs, D0.1cc and D2cc may be more meaningful parameters than V20. Taking RISI for prostate cancer as an example, the dose limits for the corresponding serial organ (rectum) are D0.1cc ≤ 200 Gy and D2cc ≤ 145 Gy^14,22^. In this study, the D0.1cc and D2cc of two patients with esophagitis were significantly higher than the average levels (higher than the upper limit of 95% CI). Although patients with esophageal fistulas did not receive higher doses than patients with grade 2 esophagitis, they had previously undergone EBRT, which might be associated with the fistula. Similar to the patients with esophageal fistulas, those with tracheal fistulas showed D0.1cc and D2cc values that were significantly higher than the average levels (higher than the upper limit of 95% CI). However, in the overall data, patients with higher doses (up to 117.22 Gy and 93.56 Gy for D0.1cc and D2cc of the trachea) showed no obvious tracheal toxicity, indicating that the dose-tolerance relationship between the esophagus and trachea cannot be summarized. Even so, we consider that in the treatment of patients whose mediastinum have received EBRT before, caution should be taken in implanting seeds near trachea and esophagus. According to the existing clinical experience, since the half-valent layer of Iodine-125 seed in tissues is 1.8 cm^23^, if the seed distance from esophagus, trachea and intestine is more than 2 cm, safety may be guaranteed, but it still needs to refer to the previous EBRT dose. Referring to the data of patients with fistula in this study, we will try our best to avoid the maximum cumulative dose (dose of EBRT and dose of seeds) of esophagus exceeding 80 Gy and trachea exceeding 90 Gy in future treatment.

For the six patients with skin and chest wall reactions, D0.1cc, D2cc, and Dmean were significantly higher than the average levels (higher than the upper limit of 95% CI). The maximum D0.1cc and D2cc were 384.75 Gy and 151.14 Gy, respectively. In addition, a chi-square test showed that high D0.1cc (>600 Gy), D2cc (>500 Gy) and D2cc (>90 Gy) of skin were associated with grade ≥2 radiation dermatitis. Despite this very high dose, no adverse reactions above grade 3 were observed, indicating acceptable skin tolerance to seed radiation.

Hemoptysis reactions were of low grade and no massive hemoptysis occurred. In this study, we only evaluated the large blood vessel radiation dose, considering vascular tolerance to be acceptable at this dosage. The maximum D0.1cc and D2cc for blood vessels were 188.81 Gy and 83.81 Gy, respectively. No damage or rupture of large blood vessels occurred. Stereotactic radiotherapy studies have shown that if the aortic dose is greater than 120 Gy, the incidence of grade 5 vascular toxicity can increase by up to 6%^24^, similar to findings for the heart and spinal cord. The maximum Dmean for the heart in this study was 20.96 Gy. The maximum D0.1cc and D2cc for the spinal cord were 606.80 Gy and 172.22 Gy, respectively. No definite toxicity or adverse effects were observed. A larger sample size and longer follow-up period are needed to confirm whether RISI is safe at such high doses.

Although the toxicities in this study were low and appeared to be acceptable, the data was retrospective and toxicities may not have been accurately captured. In addition, our follow-up period was relatively short (the median follow-up period was 11 months) may be partially due to the survival time of most patients was short because of all the patients had primary lung cancer or metastatic lung cancer of the lung, their prognosis is poor, and they received RISI as a palliative treatment. So some toxicities may not be observed. Because of these limitations, the current data are only intended for reference, and well-designed, prospective studies are needed to further clarify the safety of RISI for thoracic tumors. Since this study is aimed at radiation-related toxicity, the content of operation-related complications (such as pneumothorax, hemoptysis, hemorrhage and so on) and clinical effect were not included, we will analyze the operation-related adverse events and the effect of RISI for tumor control in the follow-on study.

## Conclusion

Because few studies have focused on non-prostate cancer, RISI use mainly depends on previous experience. Moreover, the challenges pertaining to the determination of a bioequivalent dose have not been overcome; therefore, the data from EBRT and the data for RISI in prostate cancer is not easily transferred to RISI in other tumors. In this study, the incidence of toxicity and side effects were relatively low. The correlation between dose and side effects, and the characteristics of these effects have not been fully defined. A larger sample size and longer follow-up are needed for further investigation. Given that most dosimetric parameters of side effects were higher than the upper limit of 95% CI of the overall level, we conclude that a dose within the upper limit of 95% CI is likely to be safe.

## Data Availability

The authors declare that all data supporting the findings of this study are available within the article.

## References

[1] Wang J (2018). Expert consensus statement on computed tomography-guided (125)I radioactive seeds permanent interstitial brachytherapy. Journal of cancer research and therapeutics.

[2] Zhang, F. *et al*. Chinese Expert Consensus Workshop Report: Guideline for permanent iodine-125 seed implantation of primary and metastatic lung tumors. *Thoracic Cancer* **0**, 10.1111/1759-7714.12912 (2018). 10.1111/1759-7714.12912 PMC6360234 30521144

[3] Zaorsky NG (2017). The evolution of brachytherapy for prostate cancer. Nature reviews. Urology.

[4] Zhu L (2013). An investigation of 125I seed permanent implantation for recurrent carcinoma in the head and neck after surgery and external beam radiotherapy. World journal of surgical oncology.

[5] Huang Q (2013). Computed tomographic-guided iodine-125 interstitial implants for malignant thoracic tumors. European journal of radiology.

[6] Wang H (2013). The investigation of 125I seed implantation as a salvage modality for unresectable pancreatic carcinoma. Journal of experimental & clinical cancer research: CR.

[7] Wang JJ (2009). Interstitial permanent implantation of 125I seeds as salvage therapy for re-recurrent rectal carcinoma. International journal of colorectal disease.

[8] Jiang G (2015). Computed tomography-guided iodine-125 interstitial implantation as an alternative treatment option for lung cancer. Indian journal of cancer.

[9] Li W (2015). Iodine-125 brachytherapy improved overall survival of patients with inoperable stage III/IV non-small cell lung cancer versus the conventional radiotherapy. Medical oncology (Northwood, London, England).

[10] Zhang Q, Wang DQ, Wu YF (2015). Sodium glycididazole enhances the efficacy of combined iodine-125 seed implantation and chemotherapy in patients with non small-cell lung cancer. Oncology Letters.

[11] Nath R (1995). Dosimetry of interstitial brachytherapy sources: recommendations of the AAPM Radiation Therapy Committee Task Group No. 43. American Association of Physicists in Medicine. Medical physics.

[12] Rivard MJ (2004). Update of AAPM Task Group No. 43 Report: A revised AAPM protocol for brachytherapy dose calculations. Medical physics.

[13] Davis BJ (2012). American Brachytherapy Society consensus guidelines for transrectal ultrasound-guided permanent prostate brachytherapy. Brachytherapy.

[14] Salembier C (2007). Tumour and target volumes in permanent prostate brachytherapy: a supplement to the ESTRO/EAU/EORTC recommendations on prostate brachytherapy. Radiotherapy and oncology: journal of the European Society for Therapeutic Radiology and Oncology.

[15] *Common Terminology Criteria for Adverse Events (CTCAE) version 5*.*0*, https://evs.nci.nih.gov/ftp1/CTCAE/About.html (2017).

[16] Eisenhauer EA (2009). New response evaluation criteria in solid tumours: revised RECIST guideline (version 1.1). European journal of cancer (Oxford, England: 1990).

[17] Stewart A (2016). American Brachytherapy Society consensus guidelines for thoracic brachytherapy for lung cancer. Brachytherapy.

[18] Jiang Y (2018). Side effects of CT-guided implantation of (125)I seeds for recurrent malignant tumors of the head and neck assisted by 3D printing non co-planar template. Radiation oncology (London, England).

[19] Kelly P (2010). Stereotactic body radiation therapy for patients with lung cancer previously treated with thoracic radiation. International journal of radiation oncology, biology, physics.

[20] Trovo M (2014). Stereotactic body radiation therapy for re-irradiation of persistent or recurrent non-small cell lung cancer. International journal of radiation oncology, biology, physics.

[21] Li P (2016). Single Nucleotide Polymorphisms in CBLB, a Regulator of T-Cell Response, Predict Radiation Pneumonitis and Outcomes After Definitive Radiotherapy for Non-Small-Cell Lung Cancer. Clinical lung cancer.

[22] Georg D (2014). Dosimetric considerations to determine the optimal technique for localized prostate cancer among external photon, proton, or carbon-ion therapy and high-dose-rate or low-dose-rate brachytherapy. International journal of radiation oncology, biology, physics.

[23] Nath R (1995). Dosimetry of interstitial brachytherapy sources: Recommendations of the AAPM Radiation Therapy Committee Task Group No. 43. Medical Physics.

[24] Evans JD (2013). Aortic dose constraints when reirradiating thoracic tumors. Radiother Oncol.

